# Knowledge, Attitudes, and Behaviors (KAB) of Influenza Vaccination in China: A Cross-Sectional Study in 2017/2018

**DOI:** 10.3390/vaccines8010007

**Published:** 2019-12-26

**Authors:** Xiang Ren, Elizabeth Geoffroy, Keqing Tian, Liping Wang, Luzhao Feng, Jun Feng, Ying Qin, Peng Wu, Shaosen Zhang, Mengjie Geng, Lingjia Zeng, Jianxing Yu, Benjamin J. Cowling, Zhongjie Li

**Affiliations:** 1 Division of Infectious Diseases, Key Laboratory of Surveillance and Early-Warning on Infectious Disease, Chinese Center for Disease Control and Prevention, Beijing 102206, China; renxiang@chinacdc.cn (X.R.); wanglp@chinacdc.cn (L.W.); fenglz@chinacdc.cn (L.F.); qinying@chinacdc.cn (Y.Q.); gengmj@chinacdc.cn (M.G.); lingjia.zeng@hlifetech.com (L.Z.); 2 WHO Collaborating Centre for Infectious Disease Epidemiology and Control, School of Public Health, Li Ka Shing Faculty of Medicine, The University of Hong Kong, Pokfulam 852, Hong Kong, China; pengwu@hku.hk (P.W.); bcowling@hku.hk (B.J.C.); 3 Global AIDS Interfaith Alliance, San Rafael, CA 94901, USA; emgeoffroy@thegaia.org; 4 Chinese Field Epidemiology Training Program, Chinese Center for Disease Control and Prevention, Beijing 102206, China; tiankq1989@163.com; 5 Jingzhou City Center for Disease Control and Prevention, Jingzhou 434000, China; 6 National Institute of Parasitic Disease, Chinese Center for Disease Control and Prevention, Key Laboratory of Parasite and Vector Biology, Ministry of Health, National Center for International Research on Tropical Diseases, Ministry of Science and Technology, WHO Collaborating Center for Tropical Diseases, Shanghai 200025, China; fengjun@nipd.chinacdc.cn (J.F.); zhangss@nipd.chinacdc.cn (S.Z.); 7 State Key Laboratory of Infectious Disease Prevention and Control, National Institute for Communicable Disease Control and Prevention, Chinese Center for Disease Control and Prevention, Beijing 102206, China; yujianxing@icdc.cn

**Keywords:** knowledge, attitudes, and behaviors (KAB), influenza, influenza vaccine, vaccine willingness, telephone survey, China

## Abstract

Background: This study aimed to estimate influenza-like illness (ILI) prevalence, influenza-related healthcare seeking behaviors, and willingness for vaccination. Methods: A retrospective cross-sectional study based on a random dialing telephone survey was conducted from October 2017 through March 2018 to assess influenza-like illness prevalence and vaccination willingness among different demographic groups. Results: 10,045 individuals were enrolled and completed the survey. A total of 2834 individuals (28%) self-reported that they have suffered from influenza-like illness, especially children under 15 years of age. Overall willingness for influenza vaccination in the 2018/2019 influenza season was 45% and was positively associated with higher education level, recommendation from doctors, cost-free vaccination, and vaccination campaigns with employers’ support. Hospitalization and seeking medicine from pharmacies was less frequent in urban locations. People under 15 and over 60 years of age sought medical service more frequently. Conclusions: ILI prevalence differed significantly by age and geographical location/population density. Vaccination policy for motivating key populations at highest risk to vaccinate should take into consideration the awareness-raising of vaccination benefits, barriers reduction of vaccination such as cost, and recommendation via healthcare professionals.

## 1. Introduction

The year 2018 marked the 100-year anniversary of the most severe influenza pandemic in recorded history, which infected nearly 500 million and killed an estimated of 50 million people worldwide over 2 years [1]. The World Health Organization (WHO) estimated that seasonal influenza was responsible for up to 650,000 deaths worldwide annually, and in China alone there were 456,718 reported influenza cases in 2017 with up to 92,000 annual influenza-associated respiratory deaths [2,3,4]. Seasonal epidemics and outbreaks globally caused considerable morbidity and mortality, and posed a significant threat to high-risk populations, such as pregnant women, infants, children, elderly, healthcare professionals, and patients with chronic underlying conditions (2). Furthermore, the 2017/2018 influenza season was particularly severe compared with recent years across the northern hemisphere including in China [5,6,7,8].

Vaccination is the most effective tool for influenza prevention, yet the current Expanded Program on Immunization (EPI) in China does not include the influenza vaccine; individuals are required to pay out of pocket for the influenza vaccine, and only a few locations had limited special subsidy programs for high risk groups [9]. National influenza vaccination coverage in China was just 1.5% to 2.2% between 2004 and 2014 [10]. In contrast, influenza vaccination coverage of residents over 60 years of age in 2015 was 49% in Beijing, where free influenza vaccination has been provided to residents over the age of 60 since 2007 [11]. Even more regional governments started to provide full or partial subsidy for influenza vaccines, but national influenza vaccine uptake has failed to increase substantially [10]. 

Based on a WHO fact sheet and China’s local conditions, Chinese Center for Disease Control and Prevention developed “technical guidelines for seasonal influenza vaccination in China (2018/2019)” to decrease the risk of severe infections and complications due to influenza virus infection among high risk groups. The technical guidelines recommended prioritization of seasonal influenza vaccination for children aged 6–60 months, adults over 60 years of age, persons with specific chronic diseases, healthcare workers, family members and caregivers of infants less than 6 months of age, and pregnant women or women who plan to get pregnant during the influenza season [3,12]. 

Because the sentinel surveillance system in China reports only individuals with influenza-like illness (ILI) visiting sentinel hospitals, it does not provide a full picture of ILI activities among the community. This study therefore aims to estimate the prevalence of self-reported ILI and ILI-related healthcare-seeking behaviors during the 2017/2018 influenza season in China, as well as to estimate influenza vaccination willingness for the 2018/2019 influenza season. We conducted a telephone survey in six provinces, three in northern China and three in southern China, which were sampled according to the diversified economic statuses, climate zones, and vaccine policies of provinces in China. The selected call respondents were asked about knowledge, attitudes, and behaviors relating to influenza-like illness and influenza vaccination.

## 2. Material and Methods

### 2.1. Study Design

This is a retrospective cross-sectional study using a population-based telephone survey with random digit dialing.

### 2.2. Setting

Influenza in China: China is located in the northern hemisphere. The annual seasonality of influenza A epidemics increases with latitude, whereas influenza B activity predominates in colder months throughout most of China. Influenza A epidemics peak in January–February in Northern China and April–June in the southernmost regions [13]. With reference to the WHO standard ILI case definition, body temperature ≥38 °C with either cough or sore throat was used in the survey [14].

### 2.3. Study Population and Sampling

**Figure 1:**
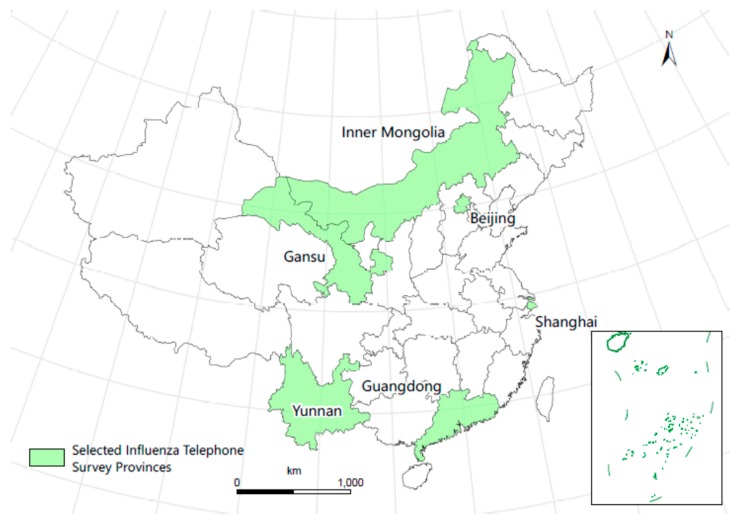
Map of the six selected provinces in the influenza-like illness survey in China, 2017/2018.

To account for differences in influenza seasonality and economic development status, six provinces, three in northern China and three in southern China, were selected for the telephone survey (Figure 1).

A representative sample population in the six provinces was reached via stratified random sampling, which was based on different ILI attack rates among different age groups. The sample size of different age groups by province was calculated on the basis of an expected ILI prevalence of 5% in adults ±2.5%, and an expected ILI prevalence of 20% in children ±5%. A minimum sample size of 9438 participants with specific amounts of each age group was required to precisely represent age-specific ILI prevalence in each location. All respondents were recruited randomly. Family members of targeted individuals under 15 years and those over 60 years of age answered the survey questions on their behalf. 

**Figure 2:**
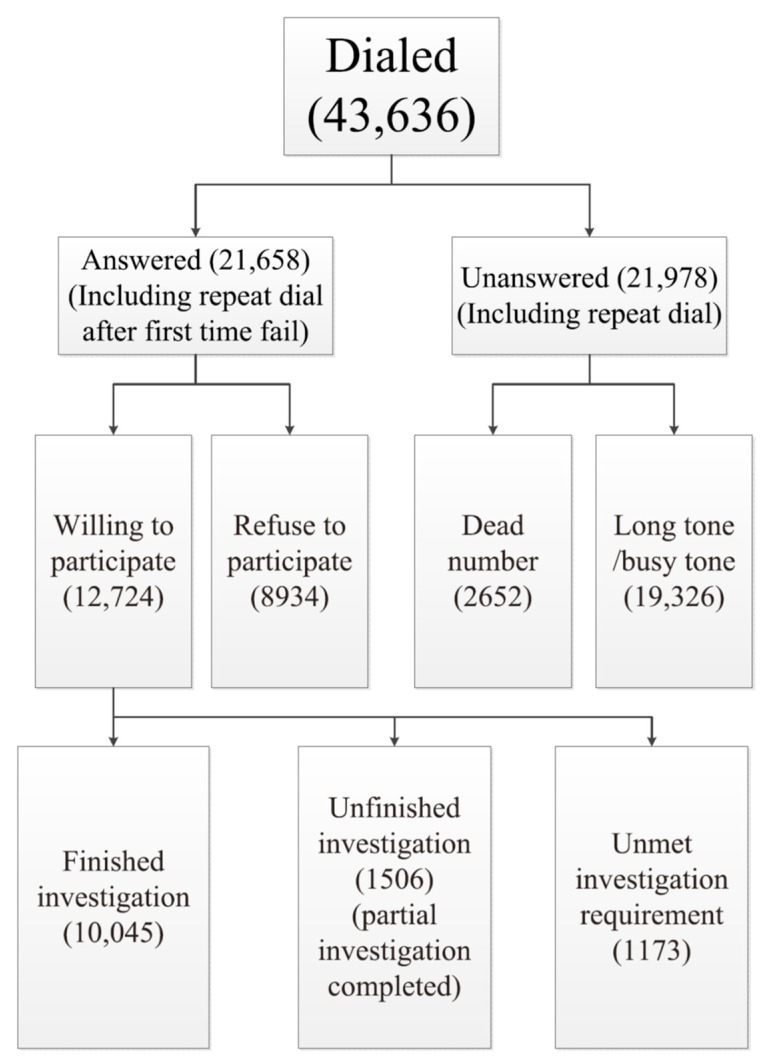
Flowchart of influenza-like illness reported in a telephone survey in six provinces of China, 2017/2018.

All survey respondents reached through telephone calls who agreed to participate and completed the telephone survey were included in the study. A total of 43,636 random telephone calls were made with an answer rate of 49.6% (21,658/43,636), of which 10,045 were willing to participate and successfully finished the questionnaire and met the requirement of five months’ residency in the study location (Figure 2).

### 2.4. Data Collection

The cross-sectional survey was performed by provincial 12320 Health Hotline Centers via the government health hotline to collect data during the October 2017 to March 2018 influenza season. The survey was conducted during March to May 2018 and the survey questionnaire included demographic information, ILI symptoms, attitude toward vaccination and protection awareness, and healthcare-seeking behaviors related to ILI symptoms. Survey data collected was double-entered into EpiData, and the data was de-identified for analysis. Phone call surveys conducted were recorded for selective examination in order to ensure the quality of the data.

### 2.5. Analysis and Statistics

Post-hoc tests, chi-square tests for goodness of fit and for trend, and logistic regression were performed using R version 3.0.1 (R Foundation for Statistical Computing, Vienna, Austria) to compare ILI rates, vaccination willingness, prevention awareness, and healthcare-seeking behaviors among different demographic groups. Significance was assigned at 5% (*p* < 0.05). 

### 2.6. Ethics Approval and Consent to Participate

Study ethics was approved the Institutional Review Board of the Chinese Center for Disease Control and Prevention (no. 201805) and the data are de-identified for protecting personal privacy. All survey respondents who agreed to participate and completed the telephone survey were included in the study.

### 2.7. Availability of Data and Materials

The datasets generated and analyzed during this study are not publicly available due to the institute’s data security and sharing policy, but are available from the corresponding author on reasonable request.

## 3. Results

Among the 10,045 enrolled individuals, 2834 self-reported suffering from influenza like illness, with a winter prevalence of 28% (2834/10,045) (Table 1). The proportion of self-reported influenza-like illness (ILI rate) in Yunnan, Inner Mongolia, and Beijing was above the overall average ILI rate (28%). In economically developed urban metropolises, such as Guangdong (mainly Guangzhou), Shanghai, and Beijing, the proportion of self-reported influenza-like illness increased from 17% to 32% as latitude increased. The influenza-like illness rate of those under age 5 (43%) and those aged 5–14 (40%) were significantly higher than the overall ILI rate. The ILI rate also increased significantly as household income increased (*p* < 0.005) (Table 1).

### 3.1. Knowledge and Attitudes toward Influenza Infection and Prevention

Among the 10,045 enrolled individuals, 75% (7564/10,045) reported knowing influenza is different from a common cold and 82% (8241/10,045) reported knowing influenza could cause severe consequences such as hospitalization and severe complications, even death. A total of 72% of enrolled individuals would recommend influenza vaccination to their family members. To be noted, individuals with awareness of a difference between influenza and common cold (odds ratio of 1.58 with 95% CI 1.35 to 1.86) and those with awareness of severe consequences caused by influenza (odds ratio of 1.70 with 95% CI 1.42 to 2.04) were found to be more likely to recommend their family members to be vaccinated for influenza in comparison with those who are not aware of these knowledge (both *p* < 0.001). In addition, people with higher education (junior college, undergraduate, and above) were found to be significantly more likely to recommend their family members to be vaccinated (*p* < 0.001), whereas household income was found to not significantly impact attitudes towards influenza vaccination. 

Prevention awareness, including hand washing, mask wearing, and self-segregation when one has ILI symptoms, was over 70%; Beijing and Shanghai were found to have the highest prevention awareness. The overall self-reported hand washing rate was found to be 79% (range of 74–83%), and hand washing awareness in urban cities was found to be significantly higher than other provinces. The overall self-reported mask wearing rate and self-segregation rate was found to be 75% (range of 64–84%) and 72% (range of 63–80%) (Table 2). Males were found to have higher prevention awareness than females, and prevention awareness increased significantly with higher income level (*p* < 0.001) and education level (*p* < 0.001). Healthcare professionals had the highest prevention awareness, whereas full-time students were found to be the least likely to be aware of the need for self-segregation (Table 2). 

### 3.2. Healthcare-Seeking Behaviors Related to Influenza Infection

Information on healthcare seeking behaviors of individuals targeted in the telephone survey who were reported to have ILI symptoms was investigated (Table 3). Important to note is that Inner Mongolia had the highest rate of healthcare-seeking behaviors for ILI symptoms (96%), whereas Beijing and Shanghai had the lowest (82% and 82%). Hospitalization and seeking medicine from a pharmacy were found to be less frequent in economically developed urban metropolises (Beijing, Shanghai, and Guangdong). The average proportion of hospitalization and seeking medicine from a pharmacy in Yunnan, Gansu, and Inner Mongolia was four times that of economically developed urban metropolises (Beijing, Shanghai, and Guangdong). Compared with other areas, individuals in Yunnan preferred a private clinic/village doctor rather than a municipal hospital for their first visit. Those under the age of 15 and over the age of 60 sought treatment more often and from outpatient/emergency departments; also, they were more frequently hospitalized. As household income increased, treatment-seeking activity decreased significantly (*p* = 0.013), and the proportion choosing a municipal hospital for their first visits increased dramatically (Table 3). 

### 3.3. Factors Related to Willingness to Be Vaccinated for Influenza

The overall willingness for influenza vaccination during the 2018/2019 influenza season was found to be 45% (range of 30–54%). Gansu has the largest proportion of individuals with the intention of vaccination against influenza, whereas individuals in Guangdong are the least likely to be vaccinated against influenza for the 2018/2019 influenza season. Individuals with higher education level were found to be more likely to be vaccinated against influenza for the 2018/2019 influenza season than those with a low education level (*p* < 0.001). Household income was found not to significantly impact one’s influenza vaccination intention for the next influenza season. Government officials and full-time students were more likely to be vaccinated against influenza than individuals of other occupation groups during the 2018/2019 influenza season (*p* < 0.001). 

**Figure 3:**
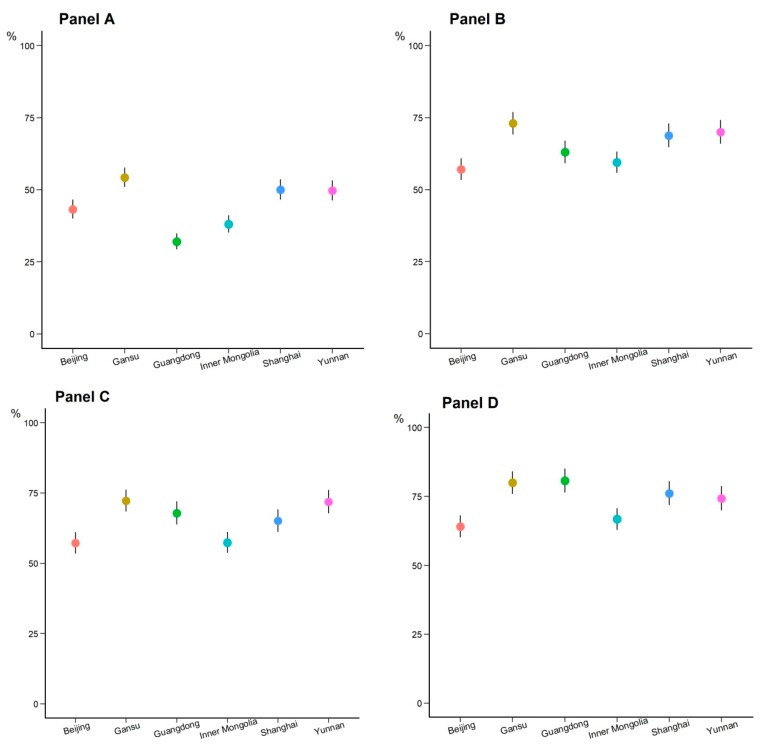

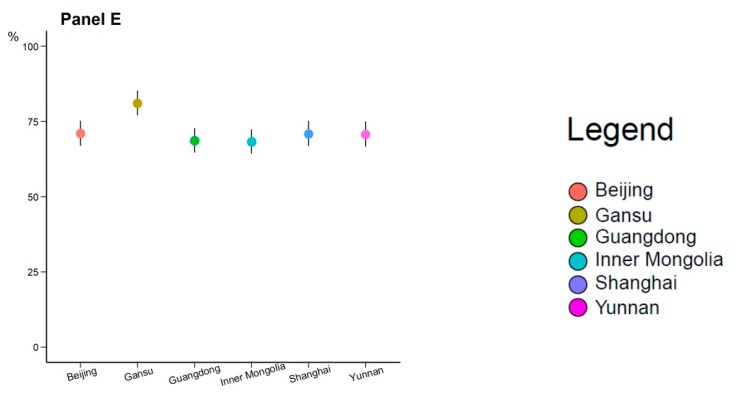
Influenza vaccination attitudes of telephone survey respondents during the October 2017 to March 2018 influenza season in six provinces of China. Panel (**A**) Willingness for influenza vaccination during the 2018/2019 influenza season in China; Panel (**B**) Willingness for influenza vaccination during the 2018/2019 influenza season on the condition that the influenza vaccine was recommended by their doctors; Panel (**C**): Willingness for influenza vaccination during the 2018/2019 influenza season on the condition that the influenza vaccine was free; Panel (**D**): Willingness for influenza vaccination during the 2018/2019 influenza season on the condition that their employer supported the influenza vaccination; Panel (**E**): Willingness for recommendation of influenza vaccination to one’s family members influenza vaccination during the 2018/2019 influenza season.

In addition, 65% of respondents self-reported with the intention of receiving an influenza vaccination on the condition that the influenza vaccine was free, 65% of respondents self-reported with the intention for influenza vaccination on the condition that the influenza vaccine was recommended by their doctors, and 74% of respondents self-reported with the intention of receiving an influenza vaccination on the condition that their employer supported the influenza vaccination (Figure 3). Recommendation from one’s doctor was found to be the most effective factor for increasing the intention for influenza vaccination (odds ratio of 6.48 with 95% CI 5.53–7.60). Cost-free vaccination also had a large effect on increasing intention for influenza vaccination (odds ratio of 5.33 with 95% CI 4.54–6.25), followed by employer’s support for influenza vaccination (odds ratio of 2.23 with 95% CI 1.85–2.68). 

## 4. Discussion

Population-based estimates of seasonal ILI are important sources of information for determining the total burden of disease. This study used population-based data from six provinces of China representing both high population density urban districts and low population density rural districts, and used a representative sample across age ranges, the influenza-like illness rate of individuals under age 14 were significantly higher than other age groups [15]. High prevention awareness overall was identified, especially among the healthcare professionals, and the varying levels of willingness to be vaccinated illustrates the need for improved communication strategies that accurately reflect an individual’s risk for contracting seasonal influenza. 

The high level of awareness of a difference between influenza and common cold and potential severe consequences caused by influenza virus infection may explain their attitudes towards influenza vaccination recommendation to their family members and the high prevention awareness. Individuals with New Rural Cooperative Medical Insurance for Rural Residents (NRCMIRR) may be reimbursed if they are vaccinated, which may account for the highest intention to vaccinate in Gansu [10]. Low willingness to vaccinate may result from individuals’ underestimation of their risk of contracting influenza and a lower ILI epidemic of the previous influenza season in their community [16]. 

Although overall prevention awareness was relatively high across all provinces studied, information, education, and communication (IEC) strategies could be targeted to the populations found to have lower awareness in this study, for instance, those with limited education and low household income who may be at increased risk for infection [17,18]. As household income does not significantly impact the influenza vaccination willingness and recommendation influenza vaccination to family members, thus IEC strategies for increasing vaccination rates should incorporate doctor’s recommendations and support from one’s employer. A policy of free influenza vaccinations for high-risk populations could also improve uptake.

Additionally, IEC campaigns that inform the public of the true estimated number of cases each year and the negative economic impacts illness has on individuals, families, and the economy as a whole may improve willingness to vaccinate, alongside expansion of free or subsidized vaccination programs. Further research is needed to estimate the cost of influenza morbidity and mortality to justify whether or not a nationwide vaccination program for key populations would improve vaccination coverage significantly [10].

The influenza seasons lasts longer with increasing latitude [19], and the influenza virus circulation patterns in the north and south of China are different [13,20]. Our results showed that the proportion of self-reported ILI in urban metropolises increases as latitude increases, which might indicate influenza transmission patterns are different in urban and rural areas as latitude increases. 

The collected data may have a selection bias and recall bias, as telephone respondents were randomly selected by the 12320 Health Hotline and were asked about self-report health information. The elderly, unemployed individuals, and those who work at home may be more likely to be involved in telephone survey. Individuals are prone to focus on health issues via the 12320 Health Hotline and thus may not be representative for all members of the population of interest. However, because of the high rate of treatment-seeking for the study population (87%) and the fact that respondents were only asked to refer back to the current influenza season, recall bias may be limited. Missing data, though minimal, and respondents’ ability to refuse participation may have impacted study findings. In addition, the individuals may have confused the influenza vaccine and haemophilus influenza type B (Hib) vaccine, which could result in higher self-reported influenza vaccination willingness. Individuals may be not ideally randomized and be skewed to those who are more often available for the 12320 Health Hotline at the time the calls were made.

The strengths of this study included random digit dialing to obtain population-based estimates of ILI by age and provided a large representative sample of the population in the six provinces. The study also adhered to the strengthening the reporting of observational studies in epidemiology (STROBE) guidelines for observational research [21].

## 5. Conclusions

ILI prevalence in different groups differed significantly by age and geographical location/population density. Vaccination willingness was positively associated with higher education level, recommendation from doctors, cost-free vaccination, and support from employers. Vaccination policy should encourage key populations at the highest risk to vaccinate, and should be accompanied by awareness- and willingness-raising campaigns that promote the benefits of vaccination, reduce barriers to vaccination such as cost, and provide information on the risk of influenza for all age groups via healthcare professionals. Additionally, campaigns should promote and educate the population on prevention strategies for women, low-income households, full-time students, and those with low levels of education.

## Figures and Tables

**Table 1:** Demographic characteristics of individuals targeted in a telephone survey in six provinces of China, October 2017 to March 2018.

Characteristic	*N*	%	Number of Self-Reported ILI * Symptoms	ILI Rate %	*p*-Value
**Province**					
Beijing	1573	16	502	32	<0.001
Shanghai	1583	16	427	27	
Guangdong	1754	17	291	17	
Inner Mongolia	1684	17	544	32	
Gansu	1859	19	525	28	
Yunnan	1592	16	545	34	
**Age**					
0–4	1977	20	842	43	<0.001
5–14	2185	22	868	40	
15–24	1657	16	330	20	
25–59	2155	21	424	20	
60+	2067	21	369	18	
Missing	4	< 1	1	25	
**Household Income (CNY)**					
Below 5,000	2137	21	582	27	0.004
5000–9999	2519	25	761	30	
10,000–19,999	2019	20	622	31	
20,000 and above	1216	12	385	32	
Missing	2154	21	484	22	
**Total**	**10,045**	**100**	**2834**	**28**	

* ILI: influenza-like illness.

**Table 2:** Influenza prevention awareness of telephone survey respondents in six provinces of China, October 2017 to March 2018.

Prevention Method	Washing Hands *				Wearing Mask *				Self-Segregation *			
	Number Responding	Number Responding Yes	%	*p*-Value	Number Responding	Number Responding Yes	%	*p*-Value	Number Responding	Number Responding Yes	%	*p*-Value
**Province**												
Beijing	1573	1306	83	<0.001	1573	1318	84	<0.001	1573	1255	80	<0.001
Shanghai	1580	1312	83		1579	1262	80		1581	1201	76	
Guangdong	1754	1423	81		1754	1125	64		1754	1231	70	
Yunnan	1553	1210	78		1559	1195	77		1522	1006	66	
Gansu	1858	1444	78		1858	1423	77		1857	1386	75	
Inner Mongolia	1678	1247	74		1679	1183	70		1679	1149	68	
**Sex**												
Female	4461	3332	75	<0.001	4466	3056	68	<0.001	4448	3002	67	<0.001
Male	5501	4581	83		5502	4421	80		5484	4201	77	
Missing	34	29	85		34	29	85		34	25	74	
**Education**												
Elementary School and below	388	243	63	<0.001	389	191	49	<0.001	381	186	49	<0.001
Middle school	974	698	72		979	610	62		971	639	66	
High school	1782	1380	77		1785	1255	70		1777	1210	68	
College and above	6674	5492	82		6672	5324	80		6660	5077	76	
Missing	178	129	72		177	126	71		177	116	66	
**Occupation**												
Farmer	385	233	61	<0.001	387	228	59	<0.001	376	219	58	<0.001
Company and enterprise employee	2978	2392	80		2980	2256	76		2976	2197	74	
Government official	1826	1470	81		1829	1441	79		1824	1383	76	
Full-time student	570	415	73		569	415	73		566	345	61	
Housewife	385	323	84		386	289	75		384	299	78	
Healthcare professional	808	735	91		808	747	92		808	698	86	
Unemployed	250	180	72		250	161	64		250	158	63	
Retired	887	690	78		887	535	60		884	580	66	
Other	1453	1141	79		1452	1094	75		1448	1034	71	
Missing	454	363	80		454	340	75		450	315	70	
**Household Income (CNY)**												
Below 5000	2110	1586	75	<0.001	2117	1495	71	<0.001	2089	1379	66	<0.001
5000–9999	2508	2017	80		2511	1926	77		2505	1867	75	
10,000–19,999	2016	1603	80		2013	1546	77		2011	1486	74	
20,000 and above	1215	1013	83		1216	977	80		1214	938	77	
Missing	2147	1723	80		2145	1562	73		2147	1558	73	
**Total**	9996	7942	79		10,002	7506	75		9966	7228	73	

* The question was phrased so that a yes response meant that if the respondent had influenza-like symptoms they would take the following precautions to prevent the spread of flu: washing hands, wearing a mask, and self-segregation.

**Table 3:** Healthcare-seeking behaviors of individuals targeted in a telephone survey in six provinces of China, October 2017 to March 2018.

Healthcare-Seeking Behaviors	Sought Treatment				First Type of Treatment *			First Visit Healthcare Facility									
	Total	Yes	%	*p*-Value	Outpatient/Emergency	Hospitalization	Medicines from Pharmacy	Private Clinic/Village Doctor	%	Community Health Service Center	%	County and District Hospital	%	Municipal Hospital	%	Do Not Remember	%
**Province**																	
Beijing	502	412	82	<0.001	367	16	55	11	3	43	10	96	23	227	55	35	8
Shanghai	427	351	82		285	11	90	10	3	28	9	87	29	160	53	17	6
Guangdong	291	255	88		221	11	47	9	4	46	19	40	17	133	56	8	3
Yunnan	545	477	88		248	47	306	158	41	82	21	31	8	92	24	22	6
Gansu	525	451	86		304	51	222	32	9	60	17	57	16	184	53	15	4
Inner Mongolia	544	524	96		353	46	222	64	15	73	17	62	14	210	48	26	6
**Age**																	
0–4	842	784	93	<0.001	646	69	211	51	7	78	11	124	17	437	61	24	3
5–14	868	781	90		600	46	266	69	10	111	16	138	20	326	48	42	6
15–24	330	272	82		186	11	137	48	21	52	23	47	20	71	31	12	5
25–59	424	324	76		185	19	162	59	24	46	18	30	12	90	36	26	10
60+	369	308	83		160	37	165	57	24	44	19	34	14	82	35	19	8
Missing	1	1	100		1		1		0	1	100		0		0		0
**Household Income (CNY)**												
Below 5000	582	522	90	0.013	325	50	262	118	27	96	22	77	18	122	28	20	5
5000–9999	761	681	89		454	52	304	68	12	109	20	93	17	243	44	38	7
10,000–19,999	622	540	87		420	44	175	34	7	60	13	95	20	261	56	20	4
20,000 and above	385	328	85		273	22	82	22	7	33	11	48	16	187	61	15	5
Missing	484	399	82		306	14	119	42	12	34	9	60	17	193	54	30	8
Total	2834	2470	87		1778	182	942	284	13	332	16	373	18	1006	47	123	6

* Multiple responses were allowed, so percentages were not reported and total will not equal the sum of the three options.
